# Obtaining filamentous fungi and lipases from sewage treatment plant residue for fat degradation in anaerobic reactors

**DOI:** 10.7717/peerj.5368

**Published:** 2018-08-14

**Authors:** Anna Cristina P. Lima, Magali C. Cammarota, Melissa L.E. Gutarra

**Affiliations:** 1 School of Chemistry, Federal University of Rio de Janeiro, Rio de Janeiro, Rio de Janeiro, Brasil; 2 Campus Xerem, Federal University of Rio de Janeiro, Duque de Caxias, Rio de Janeiro, Brasil

**Keywords:** Sewage treatment, Residues, Solid-state fermentation, Filamentous fungi, Anaerobic reactor, Lipase

## Abstract

A residue from the primary treatment of a Wastewater Treatment Plant (WWTP) was used to isolate filamentous fungi with lipase production potential. Two of the 27 isolated fungi presented high hydrolysis index and were selected for lipase production by solid-state fermentation (SSF). The fermentations were conducted at 30 °C for 48 h, with moist air circulation, using 20% (w/w) of the residue mixture with a basal medium (agroindustrial residue—babassu cake), obtaining a solid enzymatic preparation (SEP) with lipase activity of 19 U/g with the fungus identified as *Aspergillus terreus*. Scum, collected in an anaerobic reactor operating in a WWTP, was hydrolyzed with SEP and subjected to anaerobic biodegradability tests at 30 °C. Different dilutions of crude (Control) or hydrolyzed scum in raw sewage were evaluated. The dilution of 5% (v/v) of hydrolyzed scum in raw sewage proved the most adequate, as it resulted in higher methane yield compared to the raw sewage (196 and 133 mL CH_4_/g COD_added,_ respectively), without increasing the chemical oxygen demand (COD) of the treated sewage (138 and 134 mg/L). The enzymatic hydrolysis of the scum, followed by dilution in the influent sewage, is technically feasible and increases methane production in anaerobic reactors.

## Introduction

Domestic sewage consists of water (99.9%) and solids (0.1%). The solid fraction is composed of suspended and dissolved organic and inorganic materials, as well as microorganisms. The solids present in domestic sewage are composed of proteins (∼40%), carbohydrates (∼25–50%), fats (∼10%), and lower quantities of urea, surfactants, phenols, pesticides, and others (Von Sperling, 2007). Although fats represent the smallest percentage of solids in the sewage, their slow biodegradation (Perle, Kimchie & Shelef, 1995) and specific gravity which is less than that of water (generally around 0.95) causes their accumulation inside the biological reactors, and when impelled to the reactor surface by the gas bubbles, scum formation may occur (Von Sperling, 2007; Van Lier et al., 2010; Chernicharo et al., 2015).

Scum consists of floating materials scraped from the surface and contains grease, vegetable and mineral oils, animal fats, soaps, food wastes, vegetable and fruits peelings, hair, cotton, paper, cigarette tips, and other materials with density less than that of sewage. Scum is also present in grit chambers, primary and secondary settling tanks, and stabilization ponds (Von Sperling, 2007).

Since the 1980s, with the emergence of the upflow anaerobic sludge blanket (UASB) reactor, several countries in Latin America and India have adopted anaerobic technology to treat domestic sewage. Favorable climate conditions and large investments in research and development have made Latin America, notably Brazil, Colombia, and Mexico, leaders in the proper use of UASB reactors to treat domestic sewage (Chernicharo et al., 2015). A survey of 2,734 treatment facilities in Brazil, Colombia, Chile, Dominican Republic, Guatemala, and Mexico identified three major technologies to treat domestic sewage: stabilization ponds (38%), activated sludge—conventional and extended aeration (26%), and UASB reactors (17%) (Noyola et al., 2012). The use of UASB reactors in the treatment of domestic sewage is considered a mature technology in Latin America, where several large plants, treating a population equivalent to one million inhabitants, have been in operation for over 10 years (Chernicharo et al., 2015).

The scum causes several operational problems in UASB reactors by blocking pipes for feed and withdrawal of effluent; releasing unpleasant odor, attracting flies and other unwanted parasites; and interrupting the collection and removal of biogas produced, which lead to biogas losses and ruptures in the gas–liquid–solid separator system. Such problems are mitigated by the retention of the scums, which are periodically removed and sent to the post-treatment step or to the sludge discharge line (Van Lier et al., 2010).

Due to the problems associated with this type of waste (such as foul odors) and to the new solid waste management regulatory policy, introduced in 2010 in Brazil, the scum removed from anaerobic reactors needs to be stabilized and then disposed of in landfills. The per capita consumption of fats in developed countries is 50 kg/a, while in the least developed countries, it remains around 20 kg/a (Williams et al., 2012). However, with the intense degree of globalization and the tendency to follow the diet of the West, these compounds are becoming a global problem when it comes to basic sanitation, and their removal mechanisms are more complex in terms of collection and treatment. Thus, the concentration of these substances tends to increase in the sewage and on the surface of the reactors, further aggravating the problem of removal and disposal of formed scum (Williams et al., 2012; He et al., 2015).

The introduction of these lipid residues into anaerobic systems can result in increased methane production compared to proteins and carbohydrates (Domingues et al., 2015). However, the hydrolysis step of effluents with high concentration of solids is slow and considered limiting to the anaerobic digestion process (Perle, Kimchie & Shelef, 1995). Among the different pretreatments to facilitate the degradation of fats in the sewage, lipases could be an alternative, since these are efficient catalysts that specifically hydrolyze oils and greases (Donoso-Bravo & Fdz-Polanco, 2013). However, to make the use of these enzymes economically viable, it is necessary to reduce the cost of their production. The solid-state fermentation (SSF), which involves the growth of microorganisms in solid raw materials with absence or limited amount of unabsorbed water in the solid particle (Pandey, 2003), is a viable technology for enzyme production, because it is based on the use of several wastes as substrate source and can use simple production equipment and technology as the enzyme contained in the fermented solid can be applied directly only after a drying step (Leal et al., 2002; Leal et al., 2006).

Several research groups have studied mechanisms of scum formation and characterization in anaerobic reactors (Souza et al., 2006), as well as the design of devices to control scum accumulation in UASB reactors treating municipal effluents (Pereira, Celani & Chernicharo, 2009; Chernicharo et al., 2013). Such devices allow the removal of scum, which reduces operational problems in the reactors but generates more waste for disposal. After their removal, the scum is generally subjected to physical treatment (sieve and drying bed) followed by landfill disposal, with the return of the separated liquid fraction to the reactor. The option of enzymatic treatment of scum for biogas production has been considered by some authors, but only in theory, without experimental studies that prove its viability (Chernicharo et al., 2015). The present study proposes the use of scum withdrawn from the reactor to increase the organic load of the influent sewage and consequently the methane production in the reactor itself. The combined treatment (enzymatic and anaerobic biological) for fat, oil, and grease from industrial effluents has been studied (Ferreira-Leitão et al., 2017), but for the scum from domestic sewage, this has not yet been explored in the literature.

Therefore, the main objective of this work is to evaluate a combined treatment (enzymatic and anaerobic biological) for increase methane production and reduce operational problems that are associated with the formation and accumulation of scum in anaerobic reactors treating sewage. The combined treatment aims to carry out the enzymatic hydrolysis of the scum accumulated on the surface of the anaerobic reactor with enzymatic preparation containing lipases; after the hydrolysis, the hydrolyzed scum mixed with the influent sewage stream should increase the biodegradable organic load fed and consequently the methane production in the anaerobic reactor. Another aspect addressed in this work is the use of a residue from the primary treatment of a WWTP as a source of lipase-producing microorganisms and as a substrate in SSF. In addition to the economic advantage in the use of residues in the fermentation process, the large presence of microorganisms adapted to the constituents of the residue allows the selection of microbial strains with high production capacity of lipases with the characteristics desired for their application in the scum produced in the treatment plant.

## Materials and Methods

### Origin and characterization of residues to isolate filamentous fungi and SSF

The residue used as source of microorganisms and supplementary substrate in the solid-state fermentation (SSF) was collected in the primary settling tank (mix of scum and primary sludge) of a Wastewater Treatment Plant (WWTP) after drainage period to reduce moisture. Coarse and fine solids (≤1.18 mm—used in the study) fractions were separated from dried residue through sieving. The residue was stored under refrigeration (4 °C) until the time of its use.

Another residue, used as basal medium in SSF, was collected from the babassu oil industry. Called babassu cake, it was obtained after cold pressing of the babassu nut (*Orbignya oleifera*) to extract the oil. The cake was ground and sieved to separate the fraction ≤1.18 mm employed in SSF. Table 1 shows the chemical composition of both.

**Table 1:** Chemical composition of babassu cake and WWTP residue. The chemical composition of the residue used as source of microorganisms and supplementary substrate in the solid-state fermentation (SSF), collected in the primary settling tank (mix of scum and primary sludge) of a Wastewater Treatment Plant (WWTP), and of the residue used as basal medium in SSF, collected from the babassu oil industry (babassu cake) are shown.

Parameter	Babassu cake (Castro, Castilho & Freire, 2016)	WWTP residue^a^
pH	5.4	5.2
Moisture (%)	100.9	30.2 ± 2.2
O&G (mg/g)^b^	122.4	207.54 ± 7.04
Proteins (mg/g)^b^	188.0	46.21 ± 1.02
Soluble carbohydrates (mg/g)^b^	93.6	0.03 ± 0.00
Insoluble NDF (mg/g)^b^	495.9	nd
Ashes (mg/g)^b^	37.4	nd

**Notes.**

TITLE NDF neutral detergent fiber nd not determined

a Mean ± standard deviation from three samples.

b Values in mg/g dry weight.

### Isolation of filamentous fungi from WWTP residue

The filamentous fungi were isolated in Petri dishes previously prepared and sterilized, containing Potato Dextrose Agar culture medium (PDA) with final pH 5.6. With the streak-plate technique, the residue was inoculated on the plates, which were incubated for 5 days at 28 °C to grow microorganisms. This procedure was repeated until each colony was found totally isolated on the plates. Spores from each microorganism isolated were inoculated into test tubes containing slanted PDA medium and kept at 4 °C for further lipolytic activity tests.

Every three months, the same technique was used to preserve the isolated strains. After their growth, the strains were kept at 4 °C. The filamentous fungi selected for SSF was identified using capillary electrophoresis automatic sequencing analysis in the ABI 3500 Genetic Analyzer (Applied Biosystems, Foster City, CA, USA), and alignment of the nucleotide sequences was produced with the reference sequences deposited in GenBank. The results were based on Sanger sequencing.

### Qualitative evaluation of lipase production

The culture medium for the selection of filamentous fungi with lipase activity was composed of (g/100 mL): one tributyrin, two peptone, 0.1 yeast extract, 0.5 sodium chloride, and two agar (Freire et al., 1997). Spores of the isolated filamentous fungi were inoculated at the center of Petri dishes containing the culture medium, which were incubated at 30 °C for 5 days. After this period, the Hydrolysis Index (HI) was measured by the formation of a colorless halo around the colony due to the hydrolysis of the triacylglyceride and emulsion breakdown by lipase-producing microorganisms, being calculated by the ratio of total diameter (halo and colony) and colony diameter (Rocha et al., 2013).

### Solid-state fermentation of the residues and production of the solid enzymatic preparation rich in lipases

The solid-state fermentation (SSF) was conducted in a tray-type bioreactor using as culture medium a mixture of 20% (w/w) WWTP residue and babassu cake. In a cylindrical reactor (10 cm diameter), 15 g of the mixture was inoculated with spore suspension (10^7^ spores/g culture medium) and its initial moisture adjusted to 65%. Spore suspension was obtained after growth of the fungi at 28 °C for 7 days in PDA medium and scraping with sterile solution consisting of sodium phosphate buffer (50 mM, pH 7) and 0.1% (v/v) Tween 80. The number of spores was quantified by counting in the Neubauer chamber. The SSF was conducted at 30 °C in a stove with a humid air circulation for 72 h, which was monitored for moisture, pH, and lipase activity.

To quantify the lipase activity, fermented solids from a whole tray were mixed and 5 mL of sodium phosphate buffer (50 mM, pH 7) added per gram of initial residue. The enzyme was extracted on a rotary shaker at 30° C/200 rpm for 20 min. Subsequently, the blend was manually pressed to obtain crude enzyme extract, which was centrifuged at 700 g for 5 min to remove fine solids (Gombert et al., 1999).

Fermentations were performed in duplicate for the fungi *Aspergillus fumigatus* and *Aspergillus terreus*. After fermentation, the fermented mixture was dried at 40 °C for 48 h with dry air injection. The dry fermented solids were named solid enzymatic preparation (SEP—with moisture less than 10%) and stored at −20 °C until the time of use.

The enzymes from the fermented residue were extracted just to quantify the lipase activity. The obtained extract (free of cells after centrifugation) was used to analyze the activity soon after its production, while enzymatic hydrolysis experiments were conducted with the solid enzymatic preparation (SEP), dried and stored at −20 °C. This SEP contained the fermented residue, the produced enzymes, and the fungus that produced the enzymes. The use of SEP relies on the joint action of extracellular enzymes and the fungus that are active in the fermented residue.

### Enzymatic hydrolysis of scum

Six collections of material accumulated on the surface of a grit/sand separator of an Experimental Sewage Treatment Center of the Federal University of Rio de Janeiro were carried out. After removal of an aliquot for O&G quantification (in the range of 5,777 to 23,475 mg/L), this fatty material was stored at 4 °C (up to 30 days to minimize fat hydrolysis by microorganisms). To evaluate different concentrations of O&G (750, 1,500, and 3,000 mg/L), the fatty material was diluted in raw domestic sewage collected in the same place; the mixture with sewage is called “scum”. The hydrolysis tests were conducted in 500 mL flasks containing 100 mL of scum and SEP to achieve an enzymatic activity of 0.24 U/mL, incubated in a shaker at 30 °C/150 rpm. At the end of the hydrolysis, the SEP was separated by sedimentation. A blank reaction was conducted simultaneously with only 100 mL of scum, without addition of SEP.

A first hydrolysis test was conducted with 750 mg O&G/L with and without addition of 0.1 g/L sodium azide (to inhibit the activity of the microorganisms present in the scum and sewage). Aliquots of 10 mL were collected at 0, 4, 8, and 24 h of hydrolysis, and the supernatant, collected after SEP separation by sedimentation, was used to evaluate the free acid concentration and chemical oxygen demand (COD—total and soluble). Two other hydrolysis tests were conducted with 1,500 and 3,000 mg O&G/L for 4 h hydrolysis without addition of sodium azide.

### Anaerobic biodegradability tests

The anaerobic biodegradability of the scum (with 750 mg O&G/L) pre-hydrolyzed for 4 h was evaluated. The anaerobic biodegradability tests were conducted in 100 mL penicillin flasks containing 90 mL of medium. The medium contained inoculum and crude or hydrolyzed scum diluted in untreated domestic sewage to obtain different ratios (from 1 to 100% v/v). The pH of the scum was adjusted to 7.0 ± 0.2 with NaHCO_3_ before mixing with the inoculum.

Granular anaerobic sludge collected in a reactor operating in the poultry slaughtering industry was used as the inoculum, with the volume added to the penicillin flasks (15 mL) calculated for an initial concentration of 2,900 mg VSS/L. The inoculum and substrate ratio (ISR), calculated as g VS / g total COD, ranged from 0.05 to 1.26, values consistent with slow biodegradation substrates (Holliger et al., 2016).

To reduce the concentration of organic matter adsorbed into the sludge, it was incubated in the penicillin flasks for 24 h before mixing with crude or hydrolyzed scum. After addition of the scum to the sludge, the flasks were sealed with rubber stoppers and aluminum seals and incubated at 30 °C, without stirring, until stabilization of the biogas production.

Biodegradability was measured by COD removal efficiency and biogas production, as assessed by the displacement of the plunger of 60 mL graduated plastic syringes connected to the flasks until the complete stabilization of biogas production. After stabilization, the biogas produced was collected for methane analysis. The flasks were then opened to remove samples for quantification of pH and final COD. Each condition was evaluated in five replicates, and the results are presented as mean values and standard deviations.

### Analytical methods

The lipase activity was determined by titration method using olive oil as a substrate (Pandey, 2003). One unit of lipase activity being defined as the amount of enzyme that releases 1 µmol of free fatty acid per minute under the assay conditions. The lipase activity was also determined by spectrophotometric method, which is based in the formation of p-nitrophenol from the hydrolysis of p-nitrophenillaurate catalyzed by lipase (Gutarra et al., 2009). One unit of lipase activity being defined as the amount of enzyme required to hydrolyze 1 µmol of p-nitrophenillaurate per minute under the assay conditions. For pH measurement, 1 g of the fermented material was suspended in 5 mL of distilled water, the mixture was vigorously shaken for 3 min, and the pH of the supernatant measured in potentiometer. Moisture was determined on an electronic moisture analyzer Shimadzu MOC-63U. The methane content of the biogas was determined in a Varian Micro GC 4900 gas chromatograph. Other analyzes of residues characterization and monitoring of the experiments were performed according to standard methods (Greenberg, Clesceri & Eaton, 2005).

## Results

**Figure 1:**
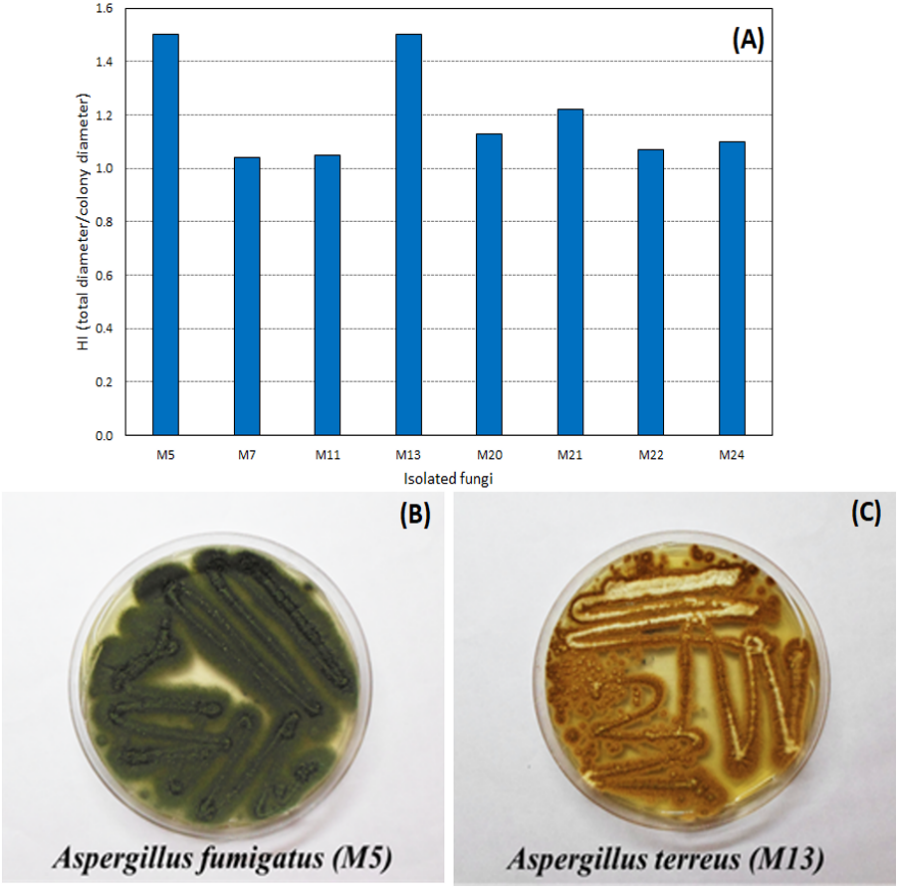
Hydrolysis Index (HI) of the tributyrin medium of the eight microorganisms isolated from the WWTP residue. Hydrolysis Index (A) and images of growth in PDA medium of the fungi with highest HI: *Aspergillus fumigatus*—M5 (B) and *Aspergillus terreus*—M13 (C).

Through the technique of isolation in Petri dishes containing only the culture medium (PDA), 27 filamentous fungi strains were found in the WWTP residue. Of the filamentous fungi isolated from the residue, eight hydrolyzed the tributyrin, forming the colorless halo and proving the lipase production capacity. The hydrolysis index (HI) ranged from 1.04 to 1.50 (Fig. 1), with two fungi, identified as *A. fumigatus* and *A. terreus*, with a highest HI (1.50).

**Figure 2:**
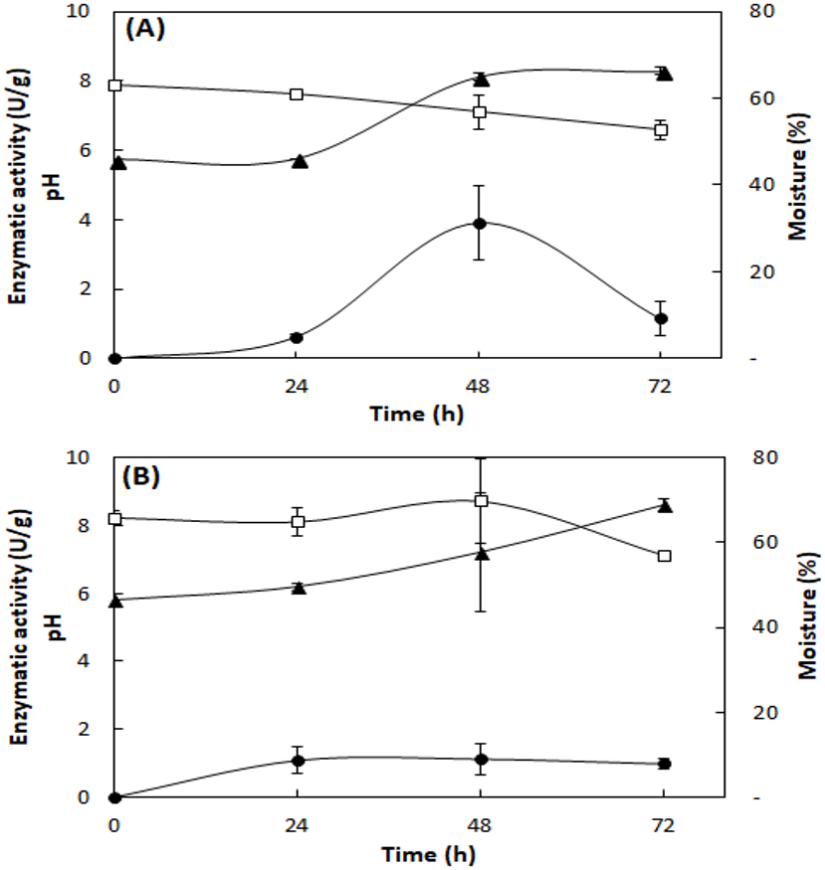
Profile of lipase activity, pH, and moisture during SSF of *A. fumigatus* (A) and *A. terreus* (B). Lipase activity (full circles), pH (full triangles), and moisture (squares). Lipase activity by the spectrophotometric method. Mean values and standard deviations from two fermentations carried out under same conditions (mixture of 20% (w/w) WWTP residue and babassu cake at 30 °C).

Figure 2 shows the lipase production, pH, and moisture profile during the fermentation of the fungi *A. fumigatus* and *A. terreus*. In the SSF with *A. fumigatus*, lipase activity presented maximum value in 48 h of fermentation (3.9 ± 1.1 U/g), which decreased at 72 h (1.2 ± 0.5 U/g), with loss of moisture and concomitant increase of pH to 8.1 (Fig. 2A). In the SSF with *A. terreus,* the moisture remained at 65% up to 48 h, decreasing to 57%, while the pH increased from an initial value of 5.8 to 8.6 at the end of the fermentation. The maximum lipase activity (1.1 ± 0.5 U/g) was reached at 48 h of fermentation, then slightly decreased (Fig. 2B).

To obtain the most efficient quantification method for lipase activity, another SSF with 48 h fermentation time was performed (Table 2), using two analytical methods (titration and spectrophotometry). Comparing the two methods to quantify lipase activity, the fermented mixture had higher activity with the titration method (18.6–21.6 U/g) than with the spectrophotometric method (2.4–5.6 U/g). The lipases produced by *A. fumigatus* lost activity during the drying process at 40 °C, while those of *A. terreus* remained active. During storage of SEP at −20 °C, pH and moisture did not change over time, while lipase activity was detected up to 14 days of storage.

**Table 2:** Lipase activity measured by titration and spectrophotometric methods, pH, and moisture at 48 h of SSF with the two fungi in the mixture of babassu cake and WWTP residue. To obtain the most efficient quantification method for lipase activity, another SSF with 48 h fermentation time was performed, using two analytical methods (titration and spectrophotometry).

Characteristics of the fermented mixture	*A. fumigatus*	*A. terreus*
Lipase activity–spectrophotometric method (U/g)	5.6 ± 0.7	2.4 ± 0.4
Lipase activity–titration method (U/g)	21.6 ± 4.4	18.6 ± 4.1
Moisture (%)	62.1 ± 1.2	60.5 ± 1.6
pH	8.0 ± 0.4	7.5 ± 0.1

**Figure 3:**
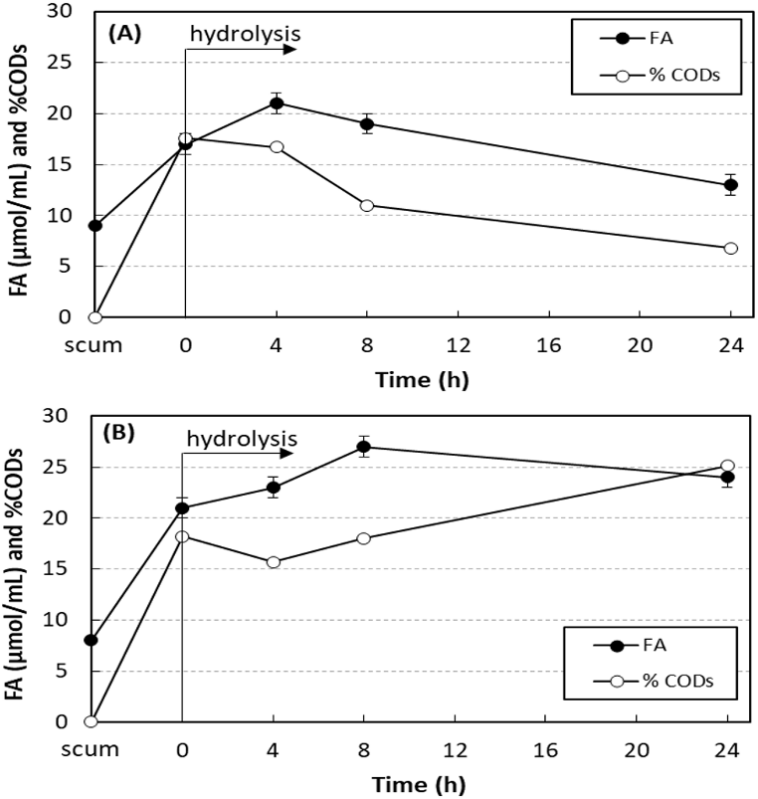
Variation in the free acid concentration (FA) and the percentage of soluble COD in relation to total COD (% CODs) in hydrolysis of scum. Enzymatic hydrolysis of the scum with 750 mg O&G/L and 0.24 U/mL of SEP produced by *A. terreus* without and with addition of sodium azide. Mean values and standard deviations of two (FA) or three (CODs) analytical replicates of each condition. Without (A) and with addition of sodium azide (B).

Figure 3 shows the results of enzymatic hydrolysis of the scum with 750 mg O&G/L and 0.24 U/mL of SEP produced by *A. terreus* without and with addition of sodium azide. The concentration of free acids (FA) and the percentage of soluble COD increased in 4 h of hydrolysis, proving the lipolytic activity of the SEP on the scum fats. Without addition of sodium azide, FA and soluble COD were reduced with the hydrolysis time. The scum prior to the addition of SEP had 8–9 µmol/mL of FA and soluble COD of 97–149 mg/L (less than 1% of total COD). Soon after the addition of SEP, both increased due to the presence of fatty acids in the SEP. At 4 h hydrolysis, without addition of sodium azide (Fig. 3A), maximum values of FA and soluble COD were achieved and increased from 17 ± 1 to 21 ± 1 µmol/mL (23% increase) and from 3,230 ± 12 to 3,920 ± 4 mg/L (21% increase), respectively. In tests performed under the same conditions, but with addition of 0.1 g/L of sodium azide (Fig. 3B), this consumption was inhibited. In this case, maximum values of FA and soluble COD were achieved after 8 h of hydrolysis, and increased from 21 ± 1 to 27 ± 0 µmol/mL (29% increase) and from 3,520 ± 11 to 5,240 ± 8 mg/L (49% increase), respectively. At 24 h of hydrolysis, 24 ± 2 µmol/mL of FA and soluble COD of 7,410 ± 18 mg/L were still observed, values 14% and 110% higher than those observed at 0 h hydrolysis.

The concentration of free acids in the tests with 1,500 mg O&G/L increased from 12 ± 1 to 16 ± 0 µmol/mL (33%), while tests in the control condition (scum only, without SEP), the value decreased from 9 ± 0 to 8 ± 0; therefore, the FA were not released. In the tests with 3,000 mg O&G/L, the FA increased from 15 to 22 µmol/mL (47%). During the 4 h of hydrolysis, volatile fatty acids (VFA) and alkalinity (Alk) increased at O&G concentrations of 750, 1,500, and 3,000 mg/L, with VFA / Alk ratios of 1.1, 1.1, and 1.4, respectively, at the end of the hydrolysis.

Total and soluble COD, biogas volume, methane percentage, total COD removal efficiency, and methane yield obtained in the biodegradability tests with different dilutions in sewage of crude scum or hydrolyzed are presented in Table 3, together with a control containing only sludge and domestic sewage. The final pH was maintained between 7.2 and 7.7 in all the tests, which are within the range of values (6.0–8.0) suitable for anaerobic digestion. The organic load introduced into the penicillin flasks increased with the scum concentration, increasing the total COD from 152 mg/L (in the sewage) to about 3,600 mg/L (with 100% crude or hydrolyzed scum). Soluble COD practically did not vary with the percentage of crude scum (from 63 to 68 mg/L) but increased considerably with the percentage of hydrolyzed scum (from 63 to 649 mg/L). In contrast, in tests conducted with hydrolyzed scum, except for the tests with 1% and 10% scum, the final COD values were lower than the initial ones and the soluble COD removals ranged from 41 to 86%. In comparison to the final COD obtained with raw sewage (134 mg/L), only the test with 5% scum maintained similar COD values, with the highest concentrations of scum showing high final COD values.

**Table 3:** Results of anaerobic biodegradability tests with different dilutions of crude or hydrolyzed scum in the sewage. Total and soluble COD, biogas volume, methane percentage, total COD removal efficiency, and methane yield obtained in the biodegradability tests with different dilutions in sewage of crude scum or hydrolyzed are presented, together with a control containing only sludge and domestic sewage.

% Scum	Initial condition		Final condition						
	CODt (mg/L)	CODs (mg/L)	pH	CODt (mg/L)	CODs (mg/L)	CODt removal (%)	Biogas (mL, 30^∘^C/1 atm)	CH_4_ (%)	MY
0–Sewage	152 ± 2	63 ± 5	7.4 ± 0.1	134 ± 13	90 ± 2	11.8 ± 8.3	6.5 ± 2.1	23.4 ± 22.2	133.4
Crude 1%	220	65	7.5 ± 0.1	172 ± 11	82 ± 23	22.0 ± 4.8	8.5 ± 1.3	27.9 ± 8.7	143.7
Hidro 1%	229	77	7.4 ± 0.1	216 ± 0	113 ± 10	5.7 ± 0.0	3.5 ± 0.7	27.3 ± 6.3	55.6
Crude 5%	494	71	7.3 ± 0.1	116 ± 19	62 ± 3	76.6 ± 3.9	8.8 ± 1.3	75.9 ± 16.9	180.3
Hidro 5%	538	134	7.2 ± 0.1	138 ± 10	80 ± 2	74.3 ±1.8	12.5 ± 0.7	63.2 ± 25.2	195.8
Crude 10%	837	79	7.4 ± 0.1	275 ± 11	241 ± 3	67.1 ± 8.5	17.7 ± 0.6	86.0 ± 6.6	242.5
Hidro 10%	924	205	7.3 ± 0.1	458 ± 71	241 ± 6	50.4 ± 7.7	17.0 ± 2.8	84.0 ± 3.6	206.1
Crude 25%	1,158	55	7.6 ± 0.1	141 ± 14	40 ± 11	81.8 ± 2.0	5.0	70.3	40.5
Hidro 25%	1,189	229	7.7 ± 0.1	232 ± 29	79 ± 4	76.6 ± 3.4	9.5 ± 0.7	78.5 ± 1.4	83.6
Crude 50%	2,181	60	7.6 ± 0.0	274 ± 23	58 ± 14	80.9 ± 1.8	7.5 ± 0.7	68.7 ± 1.7	31.5
Hidro 50%	2,242	409	7.5 ± 0.0	441 ± 30	56 ± 3	76.4 ± 1.6	17.3 ± 2.5	75.1 ± 2.2	77.3
Crude 75%	3,204	66	7.6 ± 0.1	230 ± 69	83 ± 20	88.7 ± 2.6	12.5 ± 3.5	85.8	44.6
Hidro 75%	3,296	589	7.5 ± 0.0	606 ± 94	188 ± 19	78.0 ± 0.5	20.0 ± 4.2	74.3 ± 2.0	60.1
Crude 100%	3,545	68	7.4 ± 0.1	390 ± 33	75 ± 26	83.0 ± 1.3	8.5 ± 3.5	72.8 ± 28.9	23.3
Hidro 100%	3,647	649	7.5 ± 0.0	748 ± 122	216 ± 38	75.4 ± 4.4	28.0	75.8 ± 0.9	77.6

**Notes.**

Mean values and standard deviation from 3 –5 replicates. Initial pH adjusted to 7.0 in all conditions. Final condition after 7–9 days of incubation.

TITLE MY methane yield (mL CH_4_/g total COD added). Crude or hydrolyzed scum 100% contained 750 mg O&G/L

A lag phase of 3.0 to 4.8 days in the biogas production was observed in all scum concentrations evaluated. Except in the tests with 1 to 10% scum, which had low biogas production due to the low amount of biodegradable organic matter added under these conditions, the other concentrations showed that the hydrolyzed scum presented higher accumulated biogas production (increases from 60 to 229% in relation to the production obtained with crude scum). In relation to the percentage of methane in the biogas, Table 3 shows that, except for the tests with 1% scum, all the others had methane concentrations above 70% and higher than the control with raw sewage (23.4%). The methane yield (MY) in tests with hydrolyzed scum was higher than in the tests with crude scum at dilutions of 5% and above 25%. Compared with MY obtained for the raw sewage (133.4 mL CH_4_/g COD _added_), the best results were obtained with 5% and 10% scum. Table 4 shows a summary of the results for the raw sewage and 5% hydrolyzed scum.

**Table 4:** Summary of the results obtained in the anaerobic digestion of raw sewage and 5% hydrolyzed scum.

Condition	Raw sewage	5% hydrolyzed scum
Phase lag (d)	4.4	4.3
Vmax (mL biogas/d)	3.5	7.0
Time of stabilization (d)	8	6
Final CODt (mg/L)	134 ± 13	138 ± 10
CODt removal (%)	11.8 ± 8.3	76.6 ± 3.9
% CH_4_in biogas	23.4 ± 22.2	75.9 ± 16.9
Methane yield (mL CH_4_/g COD_added_)	133.4	195.8

## Discussion

Isolation of filamentous fungi from residues with high O&G content has been used in several studies (Chaturvedi et al., 2010; Griebeler et al., 2011), due to the inductive nature of the culture medium itself for lipase production, and the adaptation of the microorganisms to the substrate, without much nutritional requirement. Species of the genus *Aspergillus*, such as those found in the WWTP residue, are studied by several research groups because they are the main agents of soil and agroindustrial residues decomposition and have potential to produce several hydrolytic enzymes (Griebeler et al., 2011; Gao et al., 2008; Pereira et al., 2014). The two fungi isolates, *A. fumigatus* and *A. terreus*, grew and produced lipase in the mixture of residues (20% w/w of WWTP residue in babassu cake), demonstrating that it has the necessary nutrients for fungi metabolism. Studies that characterize lipases produced by this fungal species found good stability at alkaline pH (Shangguan et al., 2011; Rajan & Nair, 2011). The reduction of lipase activity in the range of 48 to 72 h can be attributed to the denaturation of the enzyme caused by the reduction of moisture, the presence of proteases or even the increase of pH, considering that the strain used in this work is different from that used in other studies and can produce lipases with different characteristics. Several works developed with lipases produced by this species of fungus report that favorable conditions for higher lipase activity are pH of 5.5 to 7.5 and moisture above 70% (Sethi, Nanda & Sahoo, 2016; Hamdy & Abo-Tahon, 2012; Sethi et al., 2013). Values of pH, and especially moisture, measured in this study remained outside the values indicated in the literature, which may have led to low lipase activity. However, the range of moisture depends very much on the absorption capacity of the matrix used in the SSF. In the mixture employed in this study, the maximum water absorption capacity was 65%.

A kinetic study to evaluate the production of lipases by fungi was conducted with lipase activity determined by spectrophotometric method using a synthetic substrate. This method was employed because it is a fast and well accepted method to measure lipase activity. After the kinetic study, the lipase activity was evaluated at the time of greatest production by another quantification method, which determines, by titration, the fatty acids released from the triglycerides. This method uses a natural substrate and allows the quantification of “true” lipases (Jaeger, Dijkstra & Reetz, 1999). It was used to evaluate if the enzymes are true lipases and to determine the activity of the enzymes in triacylglycerides, as this class of substrate is probably present in the scum, validating the hydrolysis potential of the SEP. The substrate used in the spectrophotometric method was the p-nitrophenillaurate and in the titulometric method olive oil, which have different chemical structures, including the size of the carbon chains and the presence of unsaturations. The difference in lipase activity can be explained by the type of substrate used in each methodology, because olive oil (substrate used in the titrimetric method) consists of triacylglycerides, which are natural substrates of lipases. While esterases hydrolyze triglycerides made of short-chain fatty acids with less than six carbon atoms and simple esters (e.g., ethyl acetate), lipases hydrolyze water-insoluble substrates, especially triglycerides made of long-chain fatty acids such as olive oil, which has 80% oleic acid (18:1) (Bornscheuer, 2002). Therefore, the enzymes produced by both fungi can be considered “true”, because they have high activity with this substrate. The phenomenon of interfacial activation may also explain the increase of lipase activity in the titrimetric method. One of the characteristics of lipase is the increase of its activity in function of the insoluble substrates, which form emulsion. In this methodology, the olive oil is emulsified; thus, the oil-water interface can activate the enzyme, with consequent increase of its activity (Reis et al., 2009).

Lipases secreted by *A. terreus* are thermostable, so no denaturation occurred during drying (Mahmoud et al., 2015). Based on the data obtained, the SEP produced by *A. terreus* was selected for the subsequent experiments. It was stored at −20 °C for up to 14 days, and its lipase activity determined by the titration method.

The actual increase of hydrolysis/solubilization of the fat by the action of the SEP is masked due to the consumption of the soluble substances released in the hydrolysis by the microorganisms present in both the scum and the sewage used to dilute the scum (Alexandre et al., 2011; Duarte et al., 2015). As the lipases have different kinetic behaviors depending on the concentration of the substrate in the reaction medium (Contesini et al., 2010) and the scum contains high values of O&G, higher concentrations of FA and soluble COD were evaluated at 4 h of hydrolysis without addition of sodium azide.

The FA production in 4 h of hydrolysis, in the three concentrations studied, varied between 4 and 7 µmol/mL. These values may seem insignificant, but when a linear correlation between soluble COD and FA concentration (COD = 246.2 × FA, with *r*^2^ = 0.944) is used, this represents an increase in soluble COD in the range of 985–1,723 mg/L. Compared to the COD of the raw sewage used in the study (138 mg/L), there would be a 7–12 fold increase in the load added to an anaerobic reactor (considering only the soluble fraction). If this increased load is biodegradable and does not interfere with anaerobic digestion, especially in methanogenesis, the production of biogas can considerably increase.

Alkalinity (Alk) and volatile fatty acids (VFA) are important monitoring parameters in anaerobic digestion, with values of the VFA/Alk ratio >0.3 indicating process disturbances and accumulation of volatile acids (Chernicharo, 2007). Therefore, a very marked increase in VFA concentration after enzymatic hydrolysis may lead to inhibition of the anaerobic process. The increase of the acids is due to the action of the lipases on the fat, while the increase of alkalinity can be explained by the release of cations during the metabolism of other compounds (e.g., proteins, which release NH_4_^+^) by the microorganisms present in the scum (Speece, 1996). As the concentration of VFA after 4 h of hydrolysis of the scum with 750 mg O&G/L presented lower values (878 mg/L, on average, against 1,397 and 2,837 mg/L obtained with 1,500 and 3,000 mg O&G/L), this condition was selected for the following experiments, namely: dilution in sewage to 750 mg O&G/L, addition of SEP to 0.24 U/mL, 4 h hydrolysis at 30°C /150 rpm.

In the biodegradability tests with different dilutions in sewage of crude scum or hydrolyzed, the consumption of the available fraction by the sludge microorganisms, quantified as soluble COD, was observed only in some tests with addition of crude scum (removals of 12, 37, and 4% in the tests with 5, 25, and 50% scum, respectively). In the other tests with addition of crude scum, the final values were higher than the initial ones, due to the action of hydrolytic bacteria present in the sludge, which contributed to the solubilization of the particulate material of the mixtures, associated to the low substrate consumption due to the low activity of the microorganisms. However, soluble COD removals observed in tests conducted with hydrolyzed scum show that there was an increase of biodegradable organic matter after the enzymatic hydrolysis and that the microorganisms present in the sludge were able to assimilate hydrolysis products better than the original constituents of the scum. Because the hydrolysis promotes the release of organic matter easily assimilated by the microorganisms, its conversion into methane becomes more efficient (Alexandre et al., 2011). Analysis of the data presented in Table 4 indicates that 5% hydrolyzed scum would be the most adequate concentration to start the experiments of adding scum to the inflow of anaerobic reactors, because it allows higher methane production (compared to raw sewage) without damaging the final total COD of treated sewage.

## Conclusions

The residue generated in the WWTP can be used to screen lipolytic microorganisms and as a supplementary substrate in SSF to produce lipases. The SEP produced by *A. terreus* can hydrolyze O&G present in scum, increasing soluble COD. The free acids released by the enzyme in the pre-hydrolysis stage were consumed by the microbial consortium present in the anaerobic sludge. Therefore, the dilution of the hydrolyzed scum in the sewage fed to the anaerobic reactor is a technically feasible alternative to increase methane production in the sewage treatment and add value to a residue generated in the WWTP.

##  Supplemental Information

10.7717/peerj.5368/supp-1 Supplemental Information 1 Nucleotide sequences Alignment of the nucleotide sequences of fungi *A. fumigatus* and *A. terreus*.

10.7717/peerj.5368/supp-2 Supplemental Information 2 Identification of microorganisms Test results based on Sanger sequencing (*A. fumigatus*).

10.7717/peerj.5368/supp-3 Supplemental Information 3 Identification of microorganisms Test results based on Sanger sequencing—*A. terreus*.

10.7717/peerj.5368/supp-4 Supplemental Information 4 Chemical composition of STP residue Results of analyses of protein, carbohydrate, O&G, pH, and humidity of residues from Sewage Treatment Plant (STP).

10.7717/peerj.5368/supp-5 Supplemental Information 5 Monitoring of anaerobic biodegradability tests Results of chemical oxygen demand (COD), biogas volume and composition during anaerobic biodegradability tests with raw and hydrolyzed scum.

10.7717/peerj.5368/supp-6 Supplemental Information 6 Results of enzymatic hydrolysis Results of enzymatic hydrolysis tests with 750 mgL^−1^ O&G: free acids production, and COD solubilization.

10.7717/peerj.5368/supp-7 Supplemental Information 7 Results of enzymatic hydrolysis tests 3,000 mgL^−1^ O&G Results of enzymatic hydrolysis tests with 3,000 mgL^−1^ O&G: free acids production, and COD solubilization.

10.7717/peerj.5368/supp-8 Supplemental Information 8 Lipase activity Lipase activity by spectrophotometric methods, pH and moisture.

10.7717/peerj.5368/supp-9 Supplemental Information 9 Lipase activity Lipase activity by titration methods.

10.7717/peerj.5368/supp-10 Supplemental Information 10 Hydrolysis Index Results of Hydrolysis Index quantification.

10.7717/peerj.5368/supp-11 Supplemental Information 11 Profile of lipase activity Profile of lipase activity, pH, and moisture during SSF of A. fumigatus and *A. terreus*.

10.7717/peerj.5368/supp-12 Supplemental Information 12 Results of enzymatic hydrolysis 1,500 mgL^−1^ O&G Results of enzymatic hydrolysis tests with 1,500 mgL^−1^ O&G: free acids production and COD solubilization

10.7717/peerj.5368/supp-13 Supplemental Information 13 Alignment of strain MI003 with Aspergillus fumigatus sequences from Genbank

10.7717/peerj.5368/supp-14 Supplemental Information 14 Alignment of strain MI004 with Aspergillus terreus sequences from Genbank

## Additional Information and Declarations

### Data Availability

The following information was supplied regarding data availability: The raw data are provided in the Supplemental Files.
