# Supplementary material for: Obtaining filamentous fungi and lipases from sewage treatment plant residue for fat degradation in anaerobic reactors
Source: PeerJ. 2018 Aug 14;6:e5368. doi: 10.7717/peerj.5368 (PMC6097491; doi:10.7717/peerj.5368)
Supplement: Supplemental Information 1 — Alignment of the nucleotide sequences of fungi A. fumigatus and A. terreus. [file peerj-06-5368-s001.pdf]

>MI003_B sequence exported fumigatus

AGGAWCCCTAACCTGATCGAGGTCACCTTAGAAAATAAAGTTGGGTGTCGGCTGGCGCCG

GCCGGGCCTACAGAGCAGGTGACAAAGCCCCATACGCTCGAGGACCGGACGCGGTGCCGC

CGCTGCCTTTCGGGCCCGTCCCCCGGGAGAGGGGGACGGGGGCCCAACACACAAGCCGTG

CTTGAGGGCAGCAATGACGCTCGGACAGGCATGCCCCCGGAATACCAGGGGCGCATGTGC

GTTCAAAGACTCGATGATTCACTGATTCTGCAWTCACATTACTTATCSCTTTCGCTGCGT

TCTCATCGATGCCGAACAAAAATCCGTTGTTGAAGTTTWACTGATWCRAAWYCACTCAAA

>MI004_F sequence exported terreus

CSGGGWWYCAWWCCTTGATCCGAGGTCAACCTGGAAAAAAACAAGTTGCAAATAAATGCG

TCGGCGGGCGCCGGCCGGGCCTACGGAGCGGAAGACGAAGCCCCATACGCTCGAGGACCG

GACGCGGTGCCGCCGCTGCCTTTCGGGCCCGTCCCCCGGGAGCCGGGGGACGAGGGCCCA

ACACACAAGCCGGGCTTGAGGGCAGCAATGACGCTCGGACAGGCATGCCCCCCGGAATAC

CAGGGGGCGCAATGTGCGTTCAAAGACTCGATGATTCACTGAATTCTGCAATTCACATTA

GTTATCGCATTTCGCTGCGTTCTTCATCGATGCCGGAACCAAGAGATCCATTGTTGAAAG

TTTTAACTGATTGCAAAGAATCACACTCAGACTGCAAGCTTTCAGAACAGGGTTCATGTT

GGGGTCTCCGGCGGGCACGGGCCCGGGGGCGAGTCGCCCCCCGGCGGCCAGCAACGCTGG

CGGGCCCGCCGAAGCAACAAGGTACAATAGTCACGGGTGGGAGGTTGGGCCATAAAGACC

CGCACTCGGTAATGATCCTTCCGCAGGTTCACCTACGGGAAAS
