# Supplementary material for: Obtaining filamentous fungi and lipases from sewage treatment plant residue for fat degradation in anaerobic reactors
Source: PeerJ. 2018 Aug 14;6:e5368. doi: 10.7717/peerj.5368 (PMC6097491; doi:10.7717/peerj.5368)
Supplement: Supplemental Information 3 — Test results based on Sanger sequencing—A. terreus. [file peerj-06-5368-s003.pdf]

Genotyping Biotecnologia
Divisão Micro-organismos- Laboratório Central

Test Results Based on Sanger Sequencing

Identification of microorganisms -MicroID

Sample: **M5.**

Client: **Fundação Coppetec.**

Contact: **Anna Cristina P de Lima.**

Report: **MC003/2015.**

Internal code: **MC 003/15.**

Date Received: **03/30/2015**

Report Date: **04/08/2015**

Result

The sequenced microorganism shows 95% identity with the fungus *Aspergillus fumigatus.*

Interpretation

The microorganism sequence obtained was compared to a DNA database. The result of this comparison demonstrated greater identity of the sample with the fungus *Aspergillus fumigatus*.

Methodology

Automatic sequencing by capillary electrophoresis on the ABI 3500 *Genetic Analyzer* (Applied Biosystems) and alignment of the nucleotide sequences produced with the reference sequences deposited in *GenBank.*

Considerations

Although the genomic region used for identification among the species is an international standard for this analysis[1], one should not rule out the identification of more than one species per sample due to the similarity between the DNAs of the species found.

References

1) *Fungal Barcoding Consortium*et al, 2011.

Av. Deputado Dante Delmanto, 1649 - Vila Paulista – Botucatu, SP - CEP:18608-393
Tel. +55 14 3813- 5094 www.genotyping.com.br
