# Supplementary figures and images for: Obtaining filamentous fungi and lipases from sewage treatment plant residue for fat degradation in anaerobic reactors
Source: PeerJ. 2018 Aug 14;6:e5368. doi: 10.7717/peerj.5368 (PMC6097491; doi:10.7717/peerj.5368)

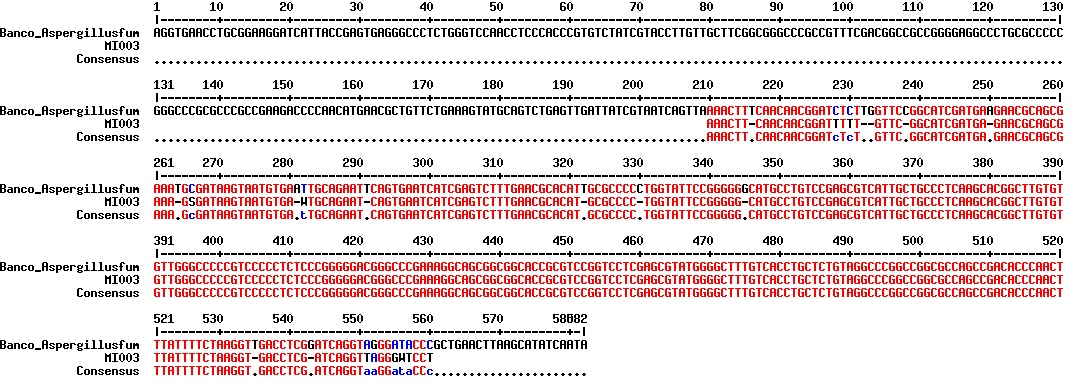

Supplement: Supplemental Information 13 [file peerj-06-5368-s013.jpg]

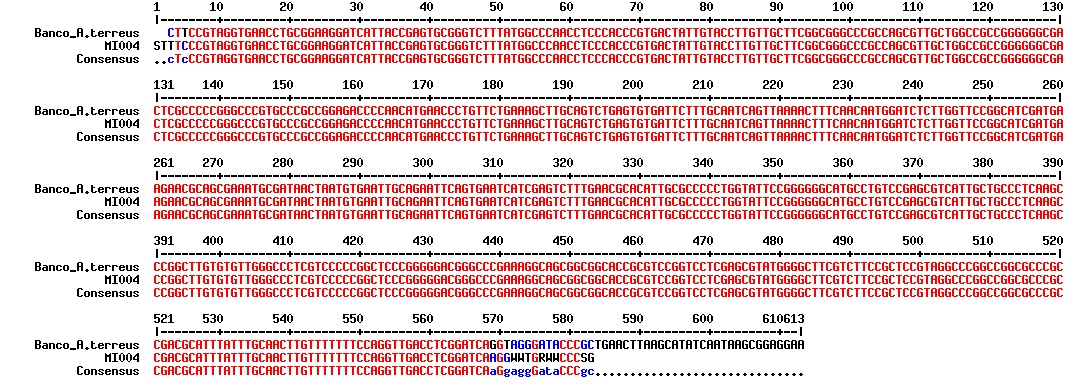

Supplement: Supplemental Information 14 [file peerj-06-5368-s014.jpg]
